# Effects of 24 Weeks of Growth Hormone Treatment on Bone Microstructure and Volumetric Bone Density in Patients with Childhood-Onset Adult GH Deficiency

**DOI:** 10.1155/2020/9201979

**Published:** 2020-03-13

**Authors:** Hongbo Yang, Kemin Yan, Yuping Xu, Linjie Wang, Qi Zhang, Fengying Gong, Huijuan Zhu, Weibo Xia, Hui Pan

**Affiliations:** ^1^Department of Endocrinology, Key Laboratory of Endocrinology of National Health Commission, The Translational Medicine Center of PUMCH, Peking Union Medical College Hospital, Chinese Academy of Medical Science and Peking Union Medical College, Beijing 100730, China; ^2^Department of Clinical Laboratory, Peking Union Medical College Hospital, Chinese Academy of Medical Sciences and Peking Union Medical College, Beijing 100730, China

## Abstract

**Objective:**

Adults with childhood-onset growth hormone deficiency (CO AGHD) have prominently impaired volumetric bone density (vBMD) and bone microarchitecture. Effects of recombinant human growth hormone (rhGH) on bone microarchitecture in CO AGHD were insufficiently evaluated. The objective of this study is to assess the effects of rhGH on bone microarchitecture and vBMD in CO AGHD patients.

**Design:**

In this single-center prospective study, nine CO AGHD patients received rhGH treatment for 24 weeks. High-resolution peripheral quantitative computerized tomography (HR-pQCT) of distal tibia and radius was performed at baseline and at the end of treatment. Main outcomes were vBMD and morphometric parameters from HR-pQCT.

**Results:**

After 24-week treatment, IGF-1 SDS gradually increased from −3.31 ± 1.56 to −1.92 ± 1.65 (*p*=0.113). Serum phosphate (1.17 ± 0.17 vs. 1.35 ± 0.18 mmol/L, *p*=0.030), alkaline phosphatase (83.6 ± 38.6 vs. 120.5 ± 63.7, *p*=0.045), and *β*-CTX (0.67 ± 0.32 vs. 1.09 ± 0.58, *p*=0.022) were significantly elevated. In distal tibia, total vBMD (200.2 ± 41.7 vs 210.3 ± 40.9 mg HA/cm^3^, *p*=0.017), cortical area (89.9 ± 17.7 vs 95.5 ± 19.9 mm^2^, *p*=0.032), and cortical thickness (0.891 ± 0.197 vs 0.944 ± 0.239 mm, *p*=0.028) were significantly improved. Trabecular area decreased from 795.3 ± 280.9 to 789.6 ± 211.4 mm^2^ (*p*=0.029). Trabecular bone volume fraction increased from 0.193 ± 0.038 to 0.198 ± 0.036 (*p*=0.027). In radius, cortical perimeter (74.1 ± 10.0 vs 75.0 ± 10.9 mm, *p*=0.034), trabecular thickness (0.208 ± 0.013 vs 0.212 ± 0.013 mm, *p*=0.008), trabecular separation (0.743 ± 0.175 vs 0.796 ± 0.199 mm, *p*=0.019), and inhomogeneity of network (Tb.1/N.SD) (0.292 ± 0.087 vs 0.317 ± 0.096 mm, *p*=0.026) were significantly improved, while trabecular number (1.363 ± 0.294 vs 1.291 ± 0.325 1/mm, *p*=0.025) decreased significantly.

**Conclusions:**

Our results provide evidence for improvement of vBMD and bone microarchitecture in AGHD patients at a relatively early stage of rhGH treatment.

## 1. Introduction

Adult growth hormone deficiency (AGHD) is a debilitating condition associated with hereditary or acquired pituitary disorders. Main clinical characteristics include low bone mineral density, sarcopenia, increased risk of metabolic syndrome, and decreased quality of life [1, 2]. Accumulating data suggested that low bone mass and increased fracture frequencies are important manifestations of AGHD [3–5]. AGHD can be divided into childhood-onset (CO AGHD) and adulthood-onset (AO AGHD). Most cases of GHD in children are idiopathic and transient, while GHD is usually permanent in multiple pituitary hormone deficiency (MPHD) [6]. The effects of rhGH replacement therapy in AGHD patients on various outcomes had been reported, and the data showed inconclusive results so far, one reason of which was selection bias especially with a mixture of CO AGHD and AO AGHD patients [7].

rhGH promotes linear growth and healthy development in childhood and plays crucial roles in normal body function in adulthood, including maintenance of bone strength, muscle volume, and other metabolic parameters [1]. There are several clinical studies evaluating the effects of rhGH on bone mineral density. In patients with both AO AGHD and CO AGHD, rhGH replacement therapy was reported to have various beneficial effects on bone mineral density (BMD) and total bone mineral content (BMC) [8–11]. Bone biopsy showed increased osteoid surface, osteoid volume, and activation frequency, but trabecular bone volume did not increase significantly [11]. In these studies, patients received 18 months to five years of rhGH treatment, and assessment of bone mineral density and bone mineral content were based on dual-energy X-ray absorptiometry (DXA) at the hip and lumbar spine. DXA evaluates 2D areal bone mineral density (aBMD) and provides no information of microarchitecture of cortical and cancellous bones. Thus, it will take a relatively long term of treatment to assess aBMD changes in AGHD patients by DXA. High-resolution peripheral quantitative computerized tomography (HR-pQCT) provides details of bone microarchitecture and volumetric bone mineral density (vBMD) [12, 13]. In our previous report, we found that young adults with childhood-onset growth hormone deficiency (CO AGHD) have prominently impaired vBMD and bone microarchitecture [14]. In the present study, effects of 24-week rhGH replacement therapy on bone microstructure and vBMD in male patients with CO AGHD were evaluated.

## 2. Subjects and Methods

### 2.1. Patients

A total of nine consecutive male patients with CO AGHD were enrolled in this prospective study from April 2017 to January 2019 in our GHD clinic at Peking Union Medical College Hospital [15]. The diagnosis of AGHD was made according to the criteria of American Endocrine Society Clinical Practice Guideline [1]. All patients had childhood-onset multiple pituitary hormone deficiency. Eight patients stopped rhGH replacement therapy since the attainment of final height. One patient did not receive rhGH treatment before. All patients sustained glucocorticoid and levothyroxine replacement since childhood. Thyroid function test was monitored to titrate LT_4_ dosage. Body weight, blood pressure, and electrolytes were monitored routinely to avoid overdose of glucocorticoids, since both overtreatment of glucocorticoids and levothyroxine can impair bone framework [16]. Oral testosterone replacement was started after completion of linear growth, and testosterone undecanoate intramuscular injection monthly was maintained in the past five years. AGHD was diagnosed with peak GH value lower than 3 *μ*g/L in insulin tolerance test.

### 2.2. Study Protocol

During 24-week treatment, the daily rhGH dose was started from subcutaneous injection of 1.0 IU per night and titrated in the event of side-effects and on an individual basis if serum IGF-1 concentration was higher than the age- and gender-adjusted reference values [1].

All patients were followed at our GHD clinic. At the start of the study and every eight weeks, physical and laboratory examinations were performed. Anthropometric parameters including height, weight, waist circumference, and hip circumference were measured with standard protocols in early morning with light clothes. BMI was calculated as weight (kg) divided by height (m) squared. Bioelectric impedance assessments (BIA) by the body composition analyzer (Tanita TBF-410, Japan) were performed in the morning after overnight fasting according to manufacturer's instructions. Fat-free mass (FFM), fat-free mass percentage (FFM%), fat mass (FM), FM percentage (FM%), and total body water (TBW) were obtained according to the body composition model. FFM index and FM index were calculated as described previously [17]. Grip strength was measured with a hand dynamometer (Jamar Plus+, Sammons Preston, Rolyon, Bolingbrook, IL, USA) on the nondominant hand, as described previously [14].

Informed consent was obtained from all patients. The study was approved by the Institutional Review Board of Peking Union Medical College Hospital (Beijing, China). All data were de-identified before analysis.

### 2.3. Biochemical Measurements

Blood samples were obtained in the morning after an overnight fasting. The serum IGF-1 level was measured with a fully automated two-site, solid-phase, chemiluminescent enzyme immunometric assay (Immulite 2000®, Siemens Healthcare Diagnostics, UK). Liver function, kidney function, HbA1c, serum calcium, serum phosphate, alkaline phosphatase (ALP), 25-hydroxyvitamin D (25 OHD), parathyroid hormone (PTH), *β*-C-terminal telopeptide region of collagen type 1 (*β*-CTX), and hormonal evaluation were all tested in the department of clinical laboratory of Peking Union Medical College Hospital by standard methods.

### 2.4. Volumetric Bone Density and Microarchitecture Assessment by HR-pQCT

High-resolution images of the nondominant distal radius and distal tibia were obtained by HR-pQCT scan (XtremeCT II scanner, ScancoMedical, Brüttisellen, Switzerland) as reported previously [14, 18]. Briefly, when a scout scan was finished, reference lines were placed at the distal end plates for both the radius and tibia. Each scan was comprised of 168 slices, corresponding to a 10.2 mm scan area, with a nominal isotropic resolution of 61 *μ*m carried out at the standard location 9.0 mm (radius) and 22.0 mm (tibia) proximal to the reference line. All scans were finished by a specified technologist and analyzed according to the standard manufacturer's method. Scans were graded for motion artifacts as described previously [19].

Bone microarchitecture parameters were obtained with the standard morphologic analysis by semiautomated software, including trabecular area (Tb.Ar), cortical area (Ct.Ar), cortical perimeter (Ct.Pm), total volume bone mineral density (Tt.vBMD), trabecular vBMD (Tb.vBMD), cortical vBMD (Ct.vBMD), trabecular separation (Tb.Sp), in-homogeneity of network (Tb.1/N.SD), cortical thickness (Ct.Th), and cortical porosity (Ct.Po) [20].

### 2.5. Statistical Analysis

Data with normal distribution are presented as mean ± standard deviation (SD), and the paired-samples *t* test was used for data analysis. Non-normal data are presented as median and ranges, and Wilcoxon signed-rank test was used for data analysis. All of the statistical computations were run using SPSS software version 22.0 for Windows (SPSS Inc., Chicago, IL, USA), and *p* < 0.05 was considered to be statistically significant.

## 3. Results

### 3.1. Demographic, Clinical, and Biochemical Characteristics

General characteristics of CO AGHD patients at baseline and after 24-week rhGH replacement therapy are shown in Table 1. A total of 9 male patients with CO AGHD with a mean age of 28 years (range, 21–51) were enrolled in this prospective study. Five patients had pituitary stalk interruption, and four patients had pituitary hypoplasia based on MRI. Eight patients stopped rhGH replacement after completion of linear growth. Duration of rhGH replacement treatment was 11.5 ± 3.1 years. The average time course since the cessation of rhGH treatment was 7.1 ± 3.3 years. One patient never accepted rhGH treatment before, and he was 51 years old at the time of the start of this study. All patients had multiple pituitary hormone deficiency and sustained glucocorticoid and levothyroxine replacement since childhood. During the 24-week study, all patients accepted 250 mg of testosterone undecanoate intramuscular injection monthly. All patients had no signs of diabetes insipidus. No clinical fracture was reported from all our patients.

After 24 weeks of treatment, there was a 0.83 increase in BMI (*p*=0.014). No significant changes were found in waist-hip ratio. Fat mass percentage decreased from 31.6% to 29.1% (*p*=0.043). Fat mass index decreased from 7.20 to 6.20 (*p*=0.012). No significant changes were found in fat-free mass index. Grip strength of nondominant hand had not been improved at the end of study (Table 1).

Endocrinological and biochemical parameters are listed in Table 2. After 24 weeks of rhGH replacement therapy, serum IGF-1 levels tended to increase from 74.8 ng/ml to 125.6 ng/ml (*p*=0.090). IGF-1 SDS increased from −3.31 ± 1.56 to −1.92 ± 1.65 (*p*=0.113). With constant replacement of levothyroxine, FT3 was stable during the study and FT4 levels decreased from 1.04 ng/dl to 0.81 ng/dl (*p*=0.024).

No routine calcium or vitamin D supplementation was prescribed during the study. Serum calcium levels remained stable after 24 weeks treatment. There were significant increases in serum phosphate (1.17 ± 0.17 vs. 1.35 ± 0.18 mmol/L, *p*=0.030), serum alkaline phosphatase levels (83.6 ± 38.6 vs. 120.5 ± 63.7, *p*=0.045), and *β*-CTX (0.67 ± 0.32 vs. 1.09 ± 0.58, *p*=0.022) after 24 weeks of treatment. Serum 25OHD decreased from 22.05 ng/ml to 17.78 ng/ml (*p*=0.032).

There was no difference in other biochemical parameters including fasting glucose, hemoglobulin, ALT, AST, albumin and uric acid at baseline and after 24 weeks of treatment.

### 3.2. Effects of 24-Week rhGH Treatment on vBMD and Microarchitecture of the Distal Tibia

**Figure 1:**
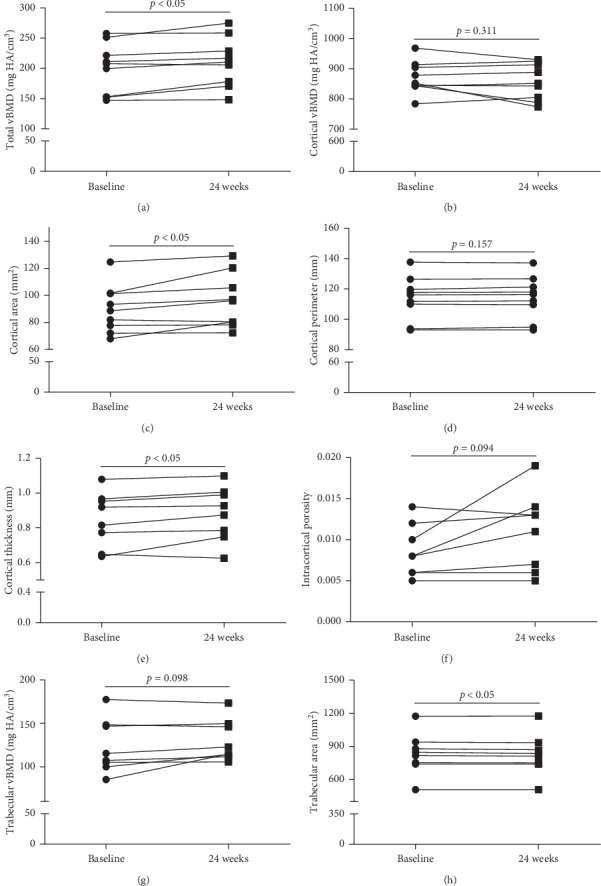
HR-pQCT parameters of the distal tibia at baseline and after 24 weeks of rhGH treatment of the patients.

Data obtained by HR-pQCT of the distal tibia are listed in Table 3. After 24-week rhGH replacement therapy, there were significant improvements of total vBMD (200.2 ± 41.7 vs 210.3 ± 40.9 mg·HA/cm^3^, *p*=0.017), trabecular bone volume fraction (0.193 ± 0.038 vs. 0.198 ± 0.036, *p*=0.027), cortical area (89.9 ± 17.7 vs 95.5 ± 19.9 mm^2^, *p*=0.032), and cortical thickness (0.891 ± 0.197 vs 0.944 ± 0.239 mm, *p*=0.028). There was significant decrease in trabecular area (795.3 ± 280.9 vs. 789.6 ± 211.4 mm^2^, *p*=0.029). No significant changes were found in cortical vBMD, cortical perimeter, and intracortical porosity of the distal tibia. No significant changes were found in trabecular vBMD, trabecular thickness, and trabecular number or trabecular separation after 24 weeks of rhGH treatment. The detailed changes of some important HR-pQCT parameters of the distal tibia at baseline and after 24 weeks of rhGH treatment are presented in Figure 1.

### 3.3. Effects of 24-Week rhGH Treatment on vBMD and Microarchitecture of the Distal Radius

**Figure 2:**
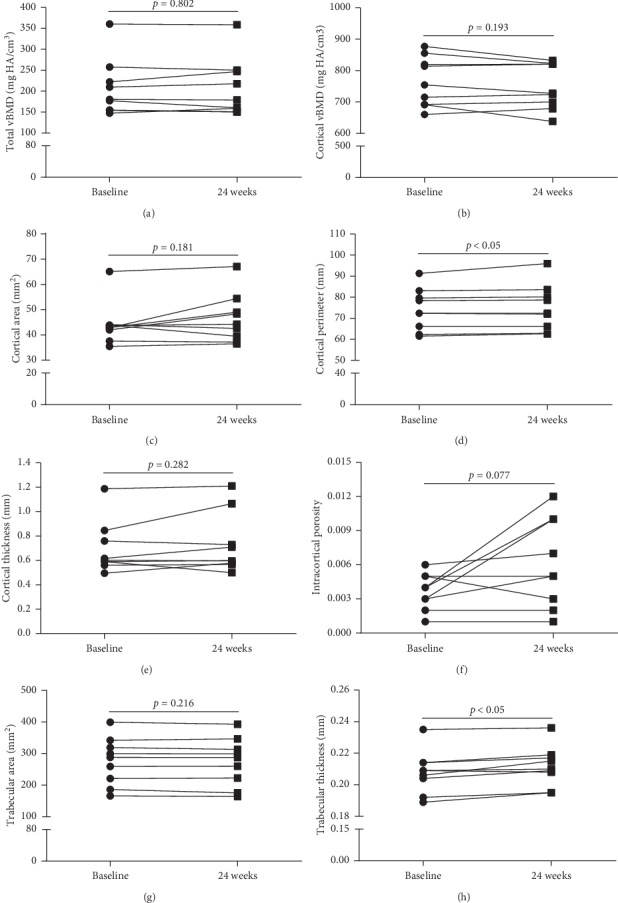
HR-pQCT parameters of the distal radius at baseline and after 24 weeks of rhGH treatment of the patients.

Data obtained by HR-pQCT of the distal radius are listed in Table 4. After 24-week rhGH replacement therapy, there were significant improvements of cortical perimeter (74.1 ± 10.0 vs 75.0 ±10.9 mm, *p* = 0.034), trabecular thickness (0.208 ± 0.013 vs 0.212 ± 0.013 mm, *p* = 0.008), and Tb.1/N.SD (0.292 ± 0.087 vs 0.317 ± 0.096 mm, *p* = 0.026). There was a significant decrease in trabecular number (1.363 ± 0.294 vs 1.291 ± 0.325 1/mm, *p* = 0.025) and increase in trabecular separation (0.743 ± 0.175 vs 0.796 ± 0.199 mm, *p* = 0.019). No significant changes were found in total vBMD, cortical area, cortical thickness, and trabecular vBMD or trabecular area after 24 weeks of rhGH treatment. The detailed changes of some important HR-pQCT parameters of the distal radius at baseline and after 24 weeks of rhGH treatment are presented in Figure 2.

## 4. Discussion

In our previous work, we found that adult male patients with CO AGHD who are no longer receiving GH replacement have abnormalities in bone microarchitecture and estimated bone strength [14]. In this pilot study, we further investigated the effects of short term rhGH replacement on bone microarchitecture in CO AGHD patients. Our results showed that rhGH significantly improved vBMD in distal tibia and bone microarchitecture in both distal tibia and radius, accompanied by increased bone resorption biomarkers in a very early stage of treatment.

GH/IGF-1 axis is a pivotal regulator of bone homeostasis and is central to the achievement of normal longitudinal bone growth and bone mass [21]. GH and IGF-1 act in an endocrine, paracrine, or autocrine fashion to regulate osteoblast, osteocyte, and osteoclast function and thus sustains normal cortical and trabecular bone properties [21, 22]. Kinetic studies demonstrated that bone calcium deposition reaches a maximum during puberty, which is approximately five times that of adulthood [23]. Thus, loss of interactions of IGF-1 and gonadosteroids and parathyroid hormone is one of the main causes of impaired peak bone mass in adults with CO AGHD [23]. Although the effect of GH and IGF-1 on bone metabolism is widely accepted, pQCT microarchitecture results in AGHD patients are inconsistent. Bone microarchitecture was reported to be marginally reduced [24] or not changed [13] in some AGHD patients. In our previous work, CO AGHD patients had significantly decreased total vBMD, cortical vBMD, trabecular vBMD, cortical area, cortical thickness, trabecular thickness, and trabecular bone volume fraction of both tibia and radius [14].

Effects of rhGH on bone mineral density in patients with AGHD have been investigated in recent decades. In both patients with CO AGHD and AO AGHD, 18 months to 5 years of rhGH replacement therapy was reported to have various beneficial effects on BMD and BMC [8–11, 25]. There were also some studies which reported that short-term rhGH treatment increased bone turnover but did not increase BMC [26]. Evaluation of BMD and BMC in all these studies is based on DXA, which does not provide details of microarchitecture of cortical and cancellous bones and could not detect subtle changes of bone in early stages of rhGH treatment. HR-pQCT thus warrants shorter-term treatment for evidences of beneficial effects of rhGH in AGHD patients with low bone mass. Our data found that rhGH is beneficial for cortical bone, including increased cortical area, cortical perimeter, and cortical thickness. These data suggested that rhGH could promote subperiosteal bone formation. At the same time, the reduction of cancellous bone area suggested cortification of cancellous bone after rhGH treatment, which is significant for maintaining the mechanical stability of bone and increasing the antifracture ability of long bone after rhGH treatment. In consistent with reports from other groups, bone-resorption biomarker *β*-CTX was increased significantly after rhGH treatment in our study [7–9]. The baseline serum vitamin D level was insufficient in our patients, and vitamin D was not supplemented during this short-term pilot study in order to avoid confounding variables to evaluate the effect of rhGH on bone volumetric density and microstructure. Further studies are required to assess the add-on effect of vitamin D in these patients.

The main limitation of this study is the small number of participants. But, there was good homogeneity in the etiology since all patients had CO AGHD due to MPHD, which is a distinct entity in AGHD and has different clinical patterns from AO AGHD. Another limitation is that there could be a selection bias in the study. Eight of the nine patients in the study undertook treatment with rhGH during childhood while the older one (51 years) never undertook treatment before enrollment. We enrolled this patient since this is a self-controlled prospective study. Another limitation is the relatively short term of treatment in our patients. We are going to follow-up these patients in long term and assess the dynamic changes of bone microarchitecture and risk of fracture in the future.

In conclusion, our findings suggest that 24 weeks of rhGH replacement induced a significant improvement in bone resorption markers and parameters of bone microarchitecture in male patients with childhood-onset adult growth hormone deficiency. CO AGHD should be recognized as a unique entity in AGHD and seamless transition management is advocated again.

## Figures and Tables

**Table 1:** Anthropometric characteristics of nine patients at baseline and after 24 weeks' treatment.

	Baseline	24-week follow-up	Changes	*p* value
Height (cm)	161.9 ± 9.0	162.6 ± 9.2	0.00 (0, 2.00)	0.050
Weight (kg)	68.5 ± 15.8	71.5 ± 17.3	2.92 ± 2.52	0.008
Waist circumference (cm)	95.5 ± 11.0	94.0 ± 12.4	−1.49 ± 2.90	0.162
Hip circumference (cm)	97.9 ± 10.5	98.4 ± 11.2	2.00 (−9.00, 4.00)	0.711
Waist-hip ratio	0.97 ± 0.04	0.95 ± 0.06	−0.19 ± 0.48	0.272
FM (kg)	18.8 ± 10.3	16.3 ± 9.8	−2.44 ± 1.29	0.013
FM (%)	31.6 ± 10.0	29.1 ± 10.0	−4.72 ± 2.83	0.043
FFM (kg)	42.7 ± 8.8	45.4 ± 8.8	4.28 ± 4.00	0.156
TBW (kg)	31.3 ± 6.4	33.2 ± 6.4	3.14 ± 2.95	0.160
BMI (kg/m^2^)	25.8 ± 4.4	26.6 ± 4.9	1.30 (−0.80, 1.50)	0.014
FMI (kg/m^2^)	7.2 ± 3.3	6.2 ± 3.2	−0.10 ± 0.51	0.012
FFMI (kg/m^2^)	17.3 ± 2.0	18.8 ± 2.6	1.50 ± 1.35	0.068
Grip strength of nondominant hand (kg)	31.9 ± 12.2	29.1 ± 14.4	0.84 ± 6.90	0.757

Data are presented as mean ± SD or median and ranges. FM, fat mass; FFM, fat-free mass; TBW, total body water; BMI, body mass index; FMI, fat mass index; FFMI, fat-free mass index.

**Table 2:** Baseline and follow-up of endocrinological and biochemical parameters.

	Baseline	24-week follow-up	Changes	*p* value
IGF-1 (ng/ml)	78.1 ± 59.3	125.6 ± 75.6	47.43 ± 62.04	0.090
IGF-1 SDS	−3.31 ± 1.56	−1.92 ± 1.65	1.38 ± 1.97	0.113
FT3 (pg/mL)	2.81 ± 0.61	2.86 ± 0.85	0.04 ± 0.75	0.880
FT4 (ng/dL)	1.04 ± 0.18	0.81 ± 0.26	−0.23 ± 0.21	0.024
Glu (mmol/L)	4.98 ± 0.62	4.73 ± 0.75	−0.24 ± 0.59	0.250
Hb (g/L)	151.9 ± 7.9	148.0 ± 9.0	−3.89 ± 8.25	0.195
ALT (U/L)	18.6 ± 7.2	19.1 ± 11.1	−3.00 (−8.00, 32.00)	0.895
AST (U/L)	24.0 ± 4.2	24.9 ± 6.0	−0.50 (−4.00, 15.00)	0.713
GGT (U/L)	19.5 ± 5.1	16.1 ± 3.9	−3.38 ± 4.44	0.069
ALP (U/L)	83.6 ± 38.6	120.5 ± 63.7	36.88 ± 42.80	0.045
Alb (g/L)	47.6 ± 3.6	47.4 ± 2.8	0.00 (−6.00, 2.00)	0.802
PA (mg/L)	263.0 ± 39.5	259.5 ± 36.5	−3.50 ± 22.90	0.769
Tbil (*μ*mol/L)	9.9 ± 3.6	13.0 ± 3.7	3.02 ± 3.09	0.022
Dbil (*μ*mol/L)	3.5 ± 1.4	4.6 ± 1.6	1.13 ± 0.88	0.008
Ca (mmol/L)	2.37 ± 0.08	2.41 ± 0.13	0.04 ± 0.11	0.365
P (mmol/L)	1.17 ± 0.17	1.35 ± 0.18	0.18 ± 0.19	0.030
25OHD (ng/ml)	22.05 ± 8.56	17.78 ± 7.72	−4.28 ± 5.34	0.032
PTH (pg/ml)	51.45 ± 19.72	45.12 ± 26.22	−6.33 ± 37.24	0.586
*β*-CTX (ng/ml)	0.67 ± 0.32	1.09 ± 0.58	0.42 ± 0.48	0.022
Cr (*μ*mol/L)	74.1 ± 8.3	77.1 ± 8.7	3.00 ± 7.50	0.264
UA (*μ*mol/L)	438.5 ± 78.6	404.4 ± 71.1	−34.13 ± 70.57	0.214

Data are presented as mean ± SD or median and ranges. IGF-1, insulin like growth factor 1; FT3, free triiodothyronine; FT4, free thyroxine; Glu, glucose; Hb, hemoglobin; ALT, alanine transaminase; AST, aspartate aminotransferase; GGT, gamma-glutamyltransferase; ALP, alkaline phosphatase; Alb, albumin; PA, prealbumin; Tbil, total bilirubin; Dbil, direct bilirubin; Ca, calcium; P, phosphate; 25OHD, 25 hydroxy-vitamin D; PTH, parathyroid hormone; *β*-CTX, *β*-C-terminal telopeptide region of collagen type 1; Cr, creatinine; UA, uric acid.

**Table 3:** Baseline and follow-up HR-pQCT parameters of the distal tibia.

	Baseline	24-week follow-up	Changes	*p* value
Total vBMD (mg HA/cm^3^)	200.2 ± 41.7	210.3 ± 40.9	10.01 ± 10.11	0.017
Cortical area (mm^2^)	89.9 ± 17.7	95.5 ± 19.9	5.60 ± 6.47	0.032
Cortical vBMD (mg HA/cm^3^)	870.3 ± 53.2	857.4 ± 60.0	8.60 (−78.60, 21.00)	0.311
Cortical perimeter (mm)	114.0 ± 14.3	114.4 ± 14.2	0.33 ± 0.64	0.157
Cortical thickness (mm)	0.891 ± 0.197	0.944 ± 0.239	0.05 ± 0.07	0.028
Intracortical porosity	0.008 ± 0.003	0.010 ± 0.005	0.001 (−0.001, 0.009)	0.094
Trabecular area (mm^2^)	795.3 ± 280.9	789.6 ± 211.4	−5.71 ± 6.44	0.029
Trabecular vBMD (mg HA/cm^3^)	121.6 ± 29.4	128.0 ± 23.0	6.37 ± 10.20	0.098
Trabecular thickness (mm)	0.245 ± 0.034	0.248 ± 0.033	0.003 ± 0.011	0.482
Trabecular number (1/mm)	1.203 ± 0.318	1.253 ± 0.311	0.012 (−0.020, 0.219)	0.102
Trabecular separation (mm)	0.878 ± 0.295	0.841 ± 0.269	−0.004 (−0.250, 0.014)	0.227
Tb.1/N.SD (mm)	0.396 (0.199, 0.964)	0.389 (0.193, 0.995)	0.001 (−0.108, 0.031)	0.597
Trabecular bone volume fraction	0.193 ± 0.038	0.198 ± 0.036	0.004 ± 0.005	0.027

Data are presented as mean ± SD or median and ranges. vBMD, volumetric bone mineral density; Tb.1/N.SD, Std. dev. of 1/Tb.N, inhomogeneity of network.

**Table 4:** Baseline and follow-up HR-pQCT parameters of the distal radius.

	Baseline	24-week follow-up	Changes	*p* value
Total vBMD (mg HA/cm^3^)	207.0 ± 68.0	208.1 ± 69.1	1.04 ± 12.08	0.802
Cortical area (mm^2^)	44.2 ± 8.4	46.5 ± 9.7	2.37 ± 4.85	0.181
Cortical vBMD (mg HA/cm^3^)	764.2 ± 79.3	751.5 ± 73.5	−12.66 ± 26.73	0.193
Cortical perimeter (mm)	74.1 ± 10.0	75.0 ± 10.9	0.50 (−0.30, 4.70)	0.034
Cortical thickness (mm)	0.602 (0.495, 1.187)	0.599 (0.500, 1.209)	0.034 ± 0.089	0.282
Intracortical porosity	0.004 ± 0.002	0.006 ± 0.004	0.002 ± 0.004	0.077
Trabecular area (mm^2^)	275.7 ± 75.5	273.5 ± 76.1	−2.13 ± 4.77	0.216
Trabecular vBMD (mg HA/cm^3^)	113.4 ± 36.4	110.2 ± 39.7	−3.20 ± 6.21	0.161
Trabecular thickness (mm)	0.208 ± 0.013	0.212 ± 0.013	0.004 ± 0.003	0.008
Trabecular number (1/mm)	1.363 ± 0.294	1.291 ± 0.325	−0.071 ± 0.078	0.025
Trabecular separation (mm)	0.743 ± 0.175	0.796 ± 0.199	0.053 ± 0.054	0.019
Tb.1/N.SD (mm)	0.292 ± 0.087	0.317 ± 0.096	0.024 ± 0.027	0.026
Trabecular bone volume fraction	0.168 ± 0.046	0.164 ± 0.050	−0.004 ± 0.007	0.134

Data are presented as mean ± SD or median and ranges. vBMD, volumetric bone mineral density; Tb.1/N.SD, Std. dev. of 1/Tb.N, inhomogeneity of network.

## Data Availability

The data used to support the findings of this study are available from the corresponding author upon request.
